# Development of a classifier to screen for severe sleep disorders in children

**DOI:** 10.3389/fped.2022.902012

**Published:** 2022-07-22

**Authors:** Mingwen Jin, Masaharu Kato, Shoji Itakura

**Affiliations:** Center for Baby Science, Doshisha University, Kyoto, Japan

**Keywords:** discriminant analysis, children, sleep problems, sleep disorder, classifier, sleep quality judgments

## Abstract

This study aimed to develop an automatic classifier for the identification of severe sleep disorders that require immediate intervention in children. Our study assessed 7,008 children (age: 0–83 months) in Japan, whose parents and nursery teachers recorded their 14-day sleep patterns. Sleep quality was assessed by pediatricians and scored as 1 (no severe sleep disorder) or 0 (severe sleep disorder). Discriminant analysis was performed for each age group using sleep quality (0 or 1) as the dependent variable and variables in the 14-day sleep log as independent variables. A stepwise method was used to select the independent variables to build the best model. The accuracy of the discriminant analysis for the age groups ranged from 71.3 to 97.3%. In summary, we developed an automatic classifier with sufficient application value to screen for severe sleep disorders in children. In the future, this classifier can be used to rapidly determine the presence or absence of severe sleep disorders in children based on their 14-day sleep logs, thus allowing immediate intervention.

## Introduction

Sleep disorders in children are common. They impair the social-emotional development of the child (1), increase his/her risk of obesity (2), and cause parental child-rearing stress (3) and maternal postpartum depression (4). As sleep problems and disorders are persistent and their frequency is high, it is important for pediatricians to screen for and identify these problems at different developmental stages (5). Notably, a large-scale study in Italy found that COVID-19 pandemic increased the incidence of sleep disorders in children aged 8–10 years; these disorders included inability to fall asleep or maintain sleep and occurrence of nightmares and/or sleep terrors (6). Owing to the persistence and high frequency of sleep disorders in children (5), it is necessary to assess children's sleep quality across various developmental stages, identify severe sleep disorders, and provide immediate intervention.

Sleep disorders in children are defined as follows: (1) sleep onset insomnia: it takes more than 60 min to fall asleep, and the time of sleep onset is after 10:30 pm; (2) sleep fragmentation: waking up more than three times during the night; (3) disturbances of continuous sleep: once awake, cannot sleep again for more than 60 min; (4) short sleep duration: total sleep duration is <8 h; and 5) variation of wake up and bed times: the variation is large, with a difference of more than 60 min (7, 8). Assessment of sleep disorders in multiple children within a specific time period is difficult owing to the time-consuming nature of the assessment.

Subjective methods of monitoring sleep status include self-reported sleepiness evaluation, life habit evaluation (9), and the Brief Infant Sleep Questionnaire-Revised (10), whereas objective methods include the use of a sleep log (11), actigraphy (12), polysomnography (13), heart rate variability analysis (14), and non-contact sleep analysis (15). Among these methods, the use of a sleep log is simple and causes minimal stress to participants. In this study, we used the sleep log observation method to determine the sleep status and sleep quality of children in a large dataset (7,008 children). The purpose of this study was to conduct a discriminant analysis of the presence or absence of severe sleep disorders in children and to develop a classifier to screen for such disorders. The classifier does not aim to classify the type of sleep disorder, which can be done using the criteria laid out in the 3rd edition of the International Classification of Sleep Disorders (16).

## Materials and methods

### Participants and procedure

Company A operates 63 childcare facilities throughout Japan. As part of its efforts to improve sleep and life rhythm, it conducted voluntary sleep surveys of children (age: 0–83 months) in childcare facilities in November 2012, September 2013, and September 2014. The parents of all participants provided informed consent. Only those who agreed to participate in the surveys were asked to record their children's 14-day sleep patterns. Data obtained from the surveys were anonymized and incorporated into the database of the Doshisha University Center for Baby Science.

### Data collection

From the database, we collected data for 7,031 children. After excluding children with incomplete data, the number of eligible study participants was 7,008. There were two types of data: those derived from the 14-day sleep log [sex (partially missing), age, record date, day of the week, wake up time, bed time, number of night wakings, self-awakening frequency, and breakfast frequency] and those pertaining to sleep quality. Sleep quality judgments were made by a pediatrician based on information from the 14-day sleep log. A, B, C, and D designations indicate good sleep (*n* = 2,391), sleep with signs of sleep disorder (*n* = 2,853), mild sleep disorder (*n* = 1,216), and severe sleep disorder that requires interventional treatment (*n* = 548), respectively. Because the purpose of this study was to develop a classifier to screen for severe sleep disorders, we divided the judgments into two categories: judgments A, B, and C (no severe sleep disorder, designated as 1) and judgment D (severe sleep disorder, designated as 0).

### Age group

Newborns are characterized by short, repeated sleep periods, regardless of their day-night rhythm. At 1 month of age, they begin differentiating between day and night. Around 3 months of age, a diurnal pattern is established, with a longer period of sleep at night and shorter naps during the day. From 6 months of age, the number of nighttime feedings decreases, and infants sleep for about 6 h at night without waking.

Infants have defined sleep stages similar to adults. By 9 months of age, 70–80% of infants can sleep through the night. At ~1 year of age, they sleep twice a day, once in the morning, and once in the afternoon. By 1.5 years of age, if they are sleeping well at night, they sleep only once a day. Napping no longer occurs between 3 and 5 years of age. By age 4, they develop a circadian rhythm, and their percentage of REM sleep at night is equal to that of adults. Naps are thought to compensate for any lack of sleep at night.

Because sleep characteristics and sleep structure differ according to age (17, 18), the children in our study were divided into nine age groups: Group 0, 0–2 months; Group 1, 3–5 months; Group 2, 6–9 months; Group 3, 10–14 months; Group 4, 15–18 months; Group 5, 19–47 months; Group 6, 48–59 months; Group 7, 60–71 months; and Group 8, 72–83 months.

### Statistical methods

IBM SPSS version 27 (19) and R-studio version 4.1.1 (20) software were used to analyze the data. Descriptive statistics, Welch's analysis of variance (ANOVA) with the Tamhene *post-hoc* test, and discriminant analysis were used in this study.

## Results

### 14-Day sleep log data

Based on the clinical findings, the following 10 variables were extracted from the 14-day sleep log and analyzed using R-studio software.

#### Wake up time

**Figure 1:**
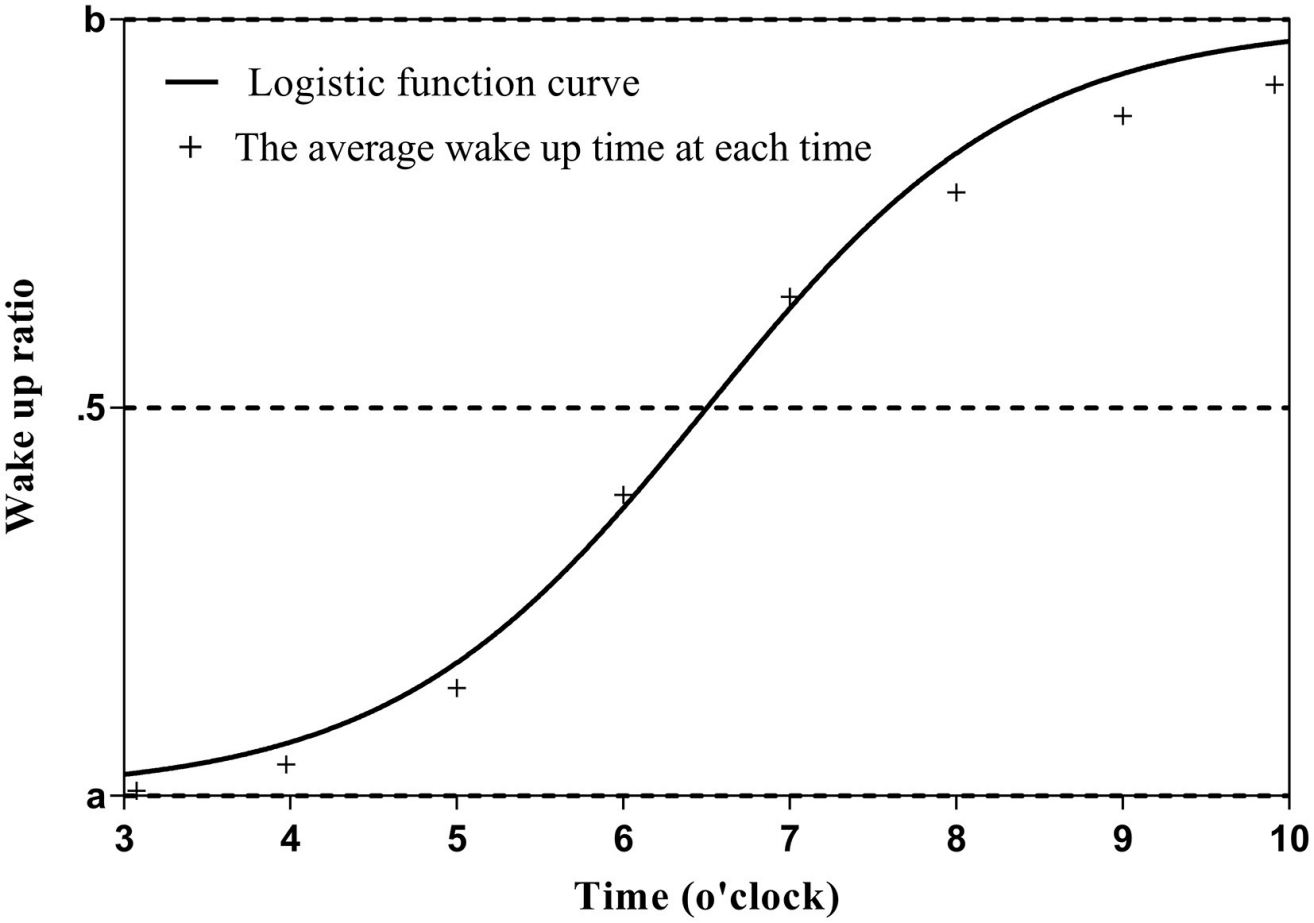
Estimation method of average waking time.

The wake up time is assumed to be between 3:00 and 10:00 in the morning, and the sleep-wake state within that time is expressed as 0 (asleep) or 1 (awake) in 5-min increments. The average sleep-wake state (sleep-wake rate) at each time (5-min increments) was calculated for the entire observation period (14 days). The data were distributed between 0 and 1, with a typical pattern of 0 from 3:00 am to 5:00 am, gradually approaching 1 from 5:00 am to 8:00 am, and 1 from 8:00 am to 10:00 am. The data were then fitted to the sigmoid function using the maximum likelihood method as follows: the closer the wake up time (x) is to 3:00 am, the closer the sleep-wake state (y) is to a; the closer the wake up time is to 10:00 am, the closer y is to b (c is the time at which the function becomes point-symmetric, and d is the slope of the function). Because y is 0 when sleeping and 1 when awake, the change in y becomes a step function and d approaches 0 if the wake up time is constant throughout the 14-day observation period. In other words, the stability of the sleep-wake rhythm can be continuously expressed by the value of d. Further, the closer d is to 0, the smaller the variation in the wake up time; c is the time when the sleep and wake states become equal (50% each) when fitted with the sigmoid function (Figure 1). The formula for the calculation of y is as follows:


(1) $$\begin{array}{l} \text{y}=\text{a}+\frac{\text{b}-\text{a}}{1+{\text{e}}^{\left(\text{c}-\text{x}\right)/\text{d}}}\; \end{array}$$

It should be noted that when fitting a sigmoid function, the maximum likelihood method often fails to converge. One of the reasons is that the function to be fitted is non-linear and thus is greatly affected by initial values. Therefore, we added a small jitter to the average sleep-wake rate obtained during the observation period, calculated it 100 times, and used the average of the obtained values as the final estimated value.

#### Wake up time variation

d in the Wake up Time Sigmoid Function.

#### Bed time

The bed time is assumed to be between 6:00 pm and 2:00 am, and the calculation method is the same as that for the wake up time. As an advantage, the maximum calculation method allows automatic determination of the wake up time and bed time even in conditions in which neither variable can be defined; such conditions include waking up once in the morning and going back to bed immediately afterward or going to bed in the evening and waking up shortly afterward. As a difference, the slope is positive for the wake up time estimate because y changes from 0 to 1 as x (the wake up time) increases, but is negative for the bed time estimate.

- Bed time variation: d in the bed time sigmoid function.
- Total sleep duration: the average of the total time spent in a sleep state in a 24-h period.
- Nocturnal sleep duration: average time spent in a sleep state between 7:00 pm and 9:00 am.
- Night wakings: mean number of awakenings during nocturnal sleep.
- Evening nap: mean number of sleep states at 3:00 and 7:00 pm.
- Breakfast: mean number of times breakfast was eaten.
- Self-awake: mean number of times children woke up by themselves.

Descriptive statistics were calculated using SPSS version 27 software. The descriptive statistics for the 10 variables are presented in Table 1.

**Table 1:** Descriptive statistics of the various variables.

**Variables**	**Min**.	**Max**.	**M**	**SD**	**Ske**.	**Kur**.
Wake up time	4.23	10.26	6.84	0.58	0.17	0.74
Wake up time variation	0.00	1.00	0.26	0.16	1.12	3.37
Bed time	18.70	1.45	21.44	0.69	0.11	0.97
Bed time variation	−1.00	0.01	−0.27	0.17	−1.20	3.20
Total sleep duration	7.00	18.21	11.27	0.84	0.04	1.32
Nocturnal sleep duration	5.25	11.88	9.18	0.58	−0.04	1.09
Night wakings	0.00	5.31	0.14	0.38	5.34	37.86
Evening nap	0.00	1.62	0.11	0.15	2.69	11.43
Breakfast	0.00	1.00	0.94	0.18	−4.14	17.41
Self-awake	0.00	1.00	0.69	0.31	−0.66	−0.76


### Sleep characteristics of different age groups

**Figure 2:**
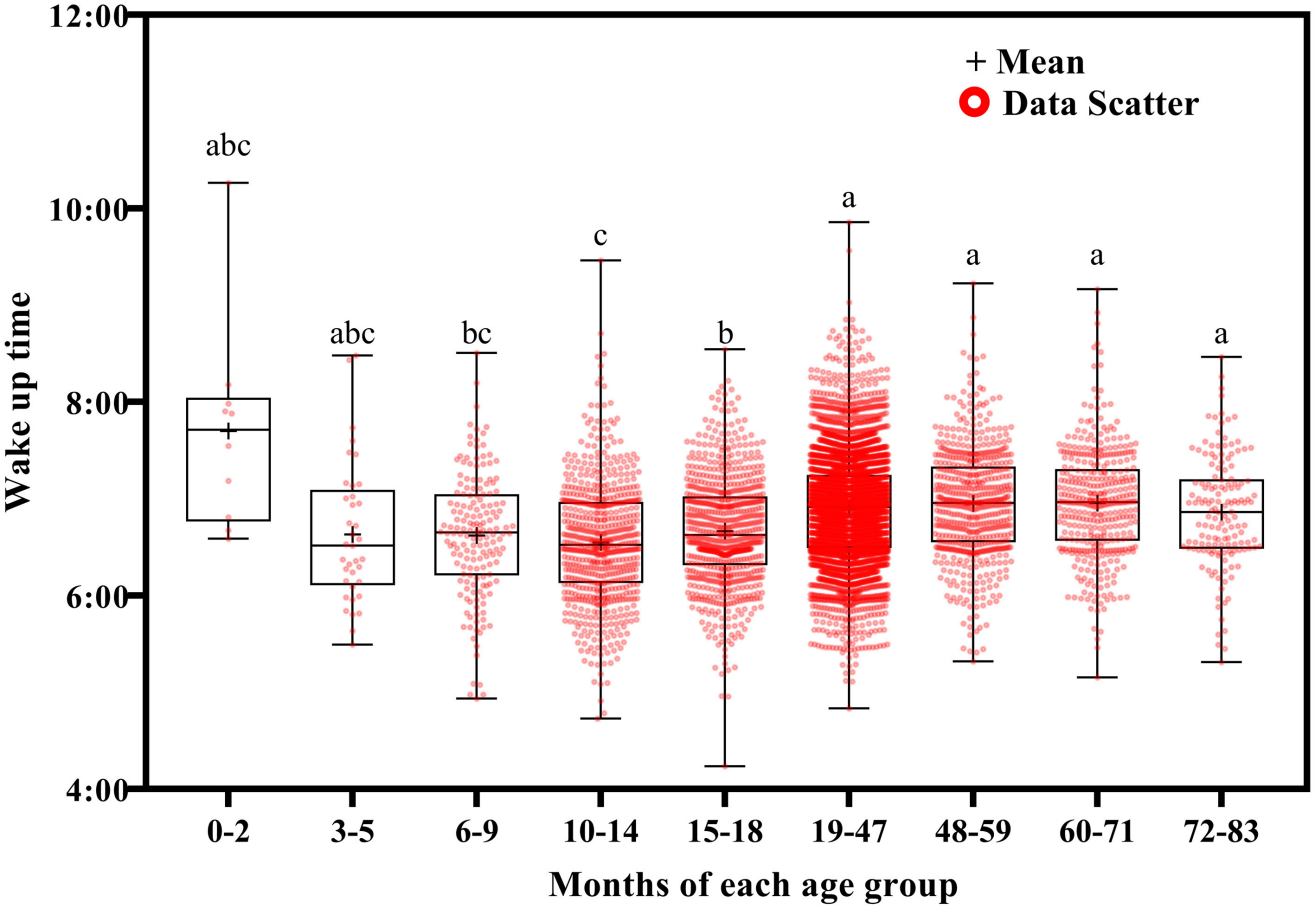
Results of the one-way ANOVA for wake up time stratified by age.

**Figure 3:**
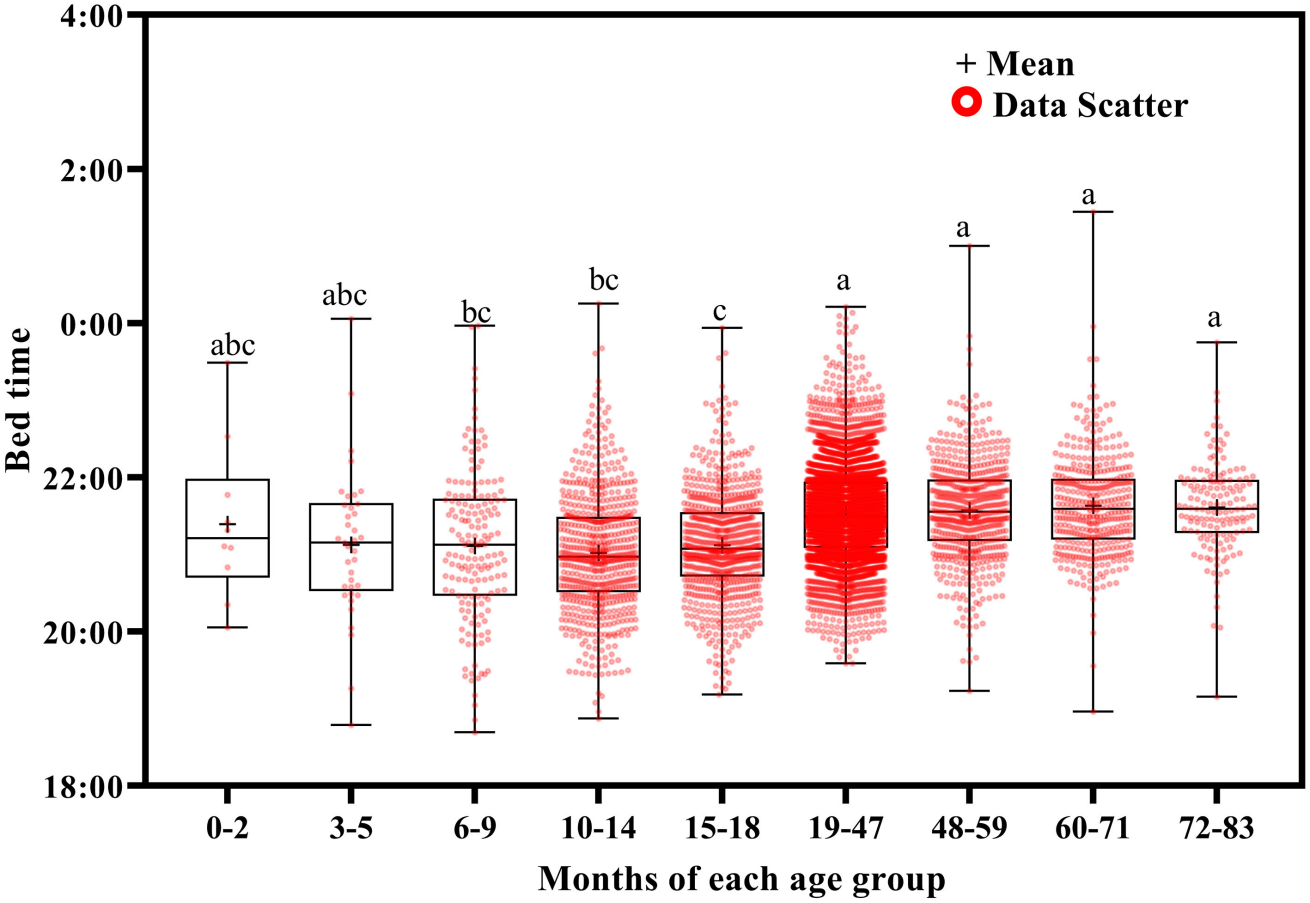
Results of the one-way ANOVA for bed time stratified by age.

**Figure 4:**
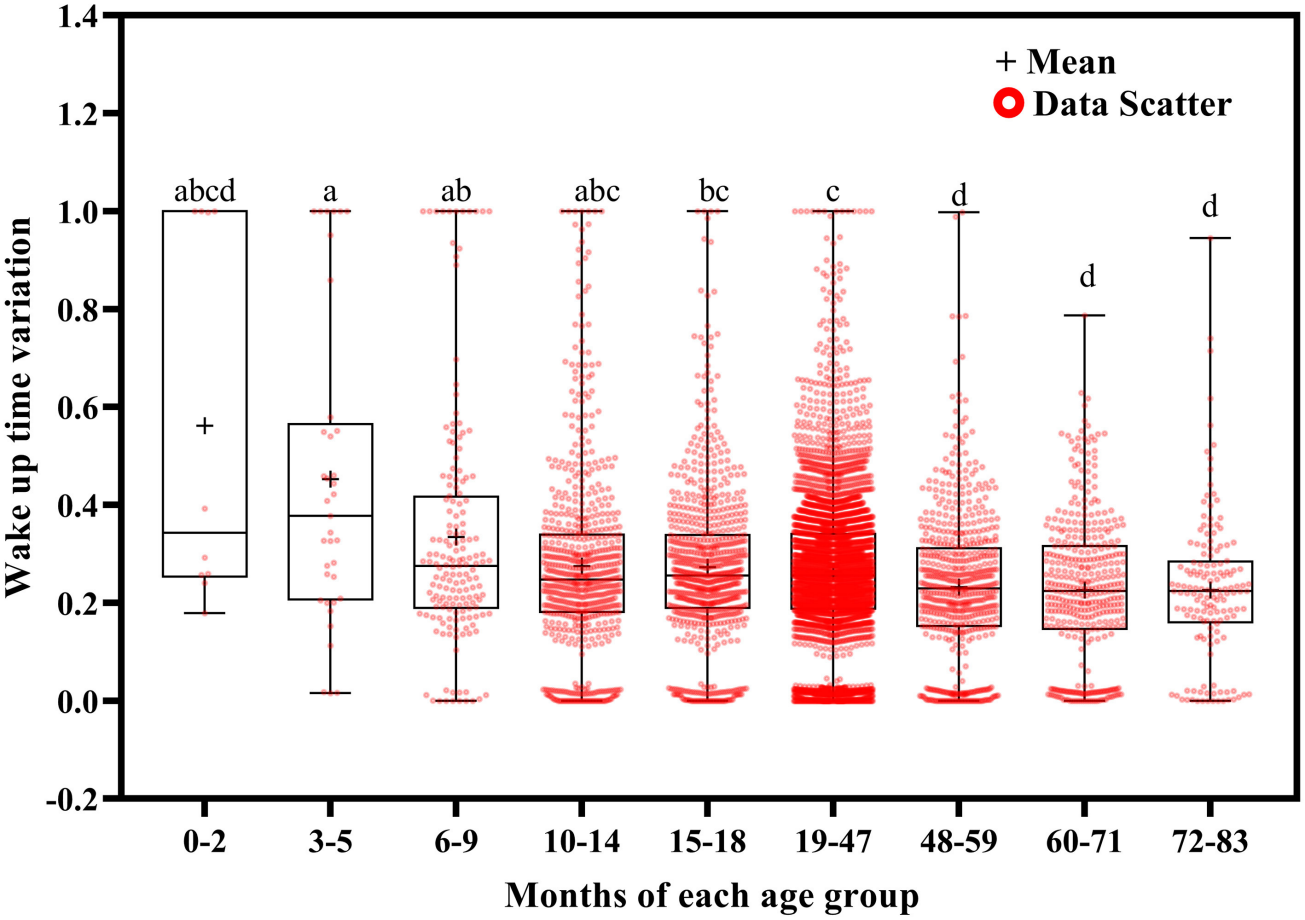
Results of the one-way ANOVA for wake up time variation stratified by age.

**Figure 5:**
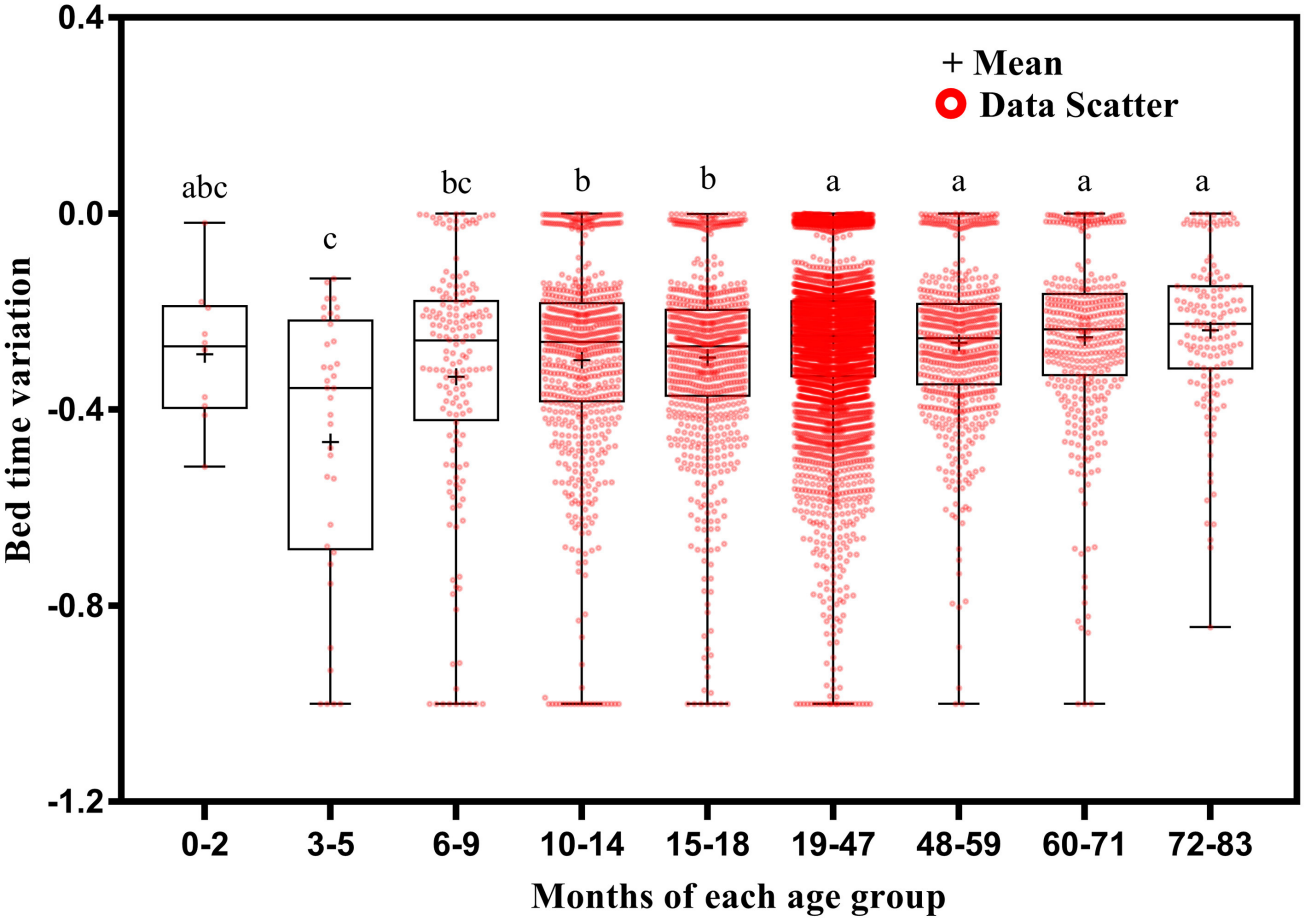
Results of the one-way ANOVA for bed time variation stratified by age.

**Figure 6:**
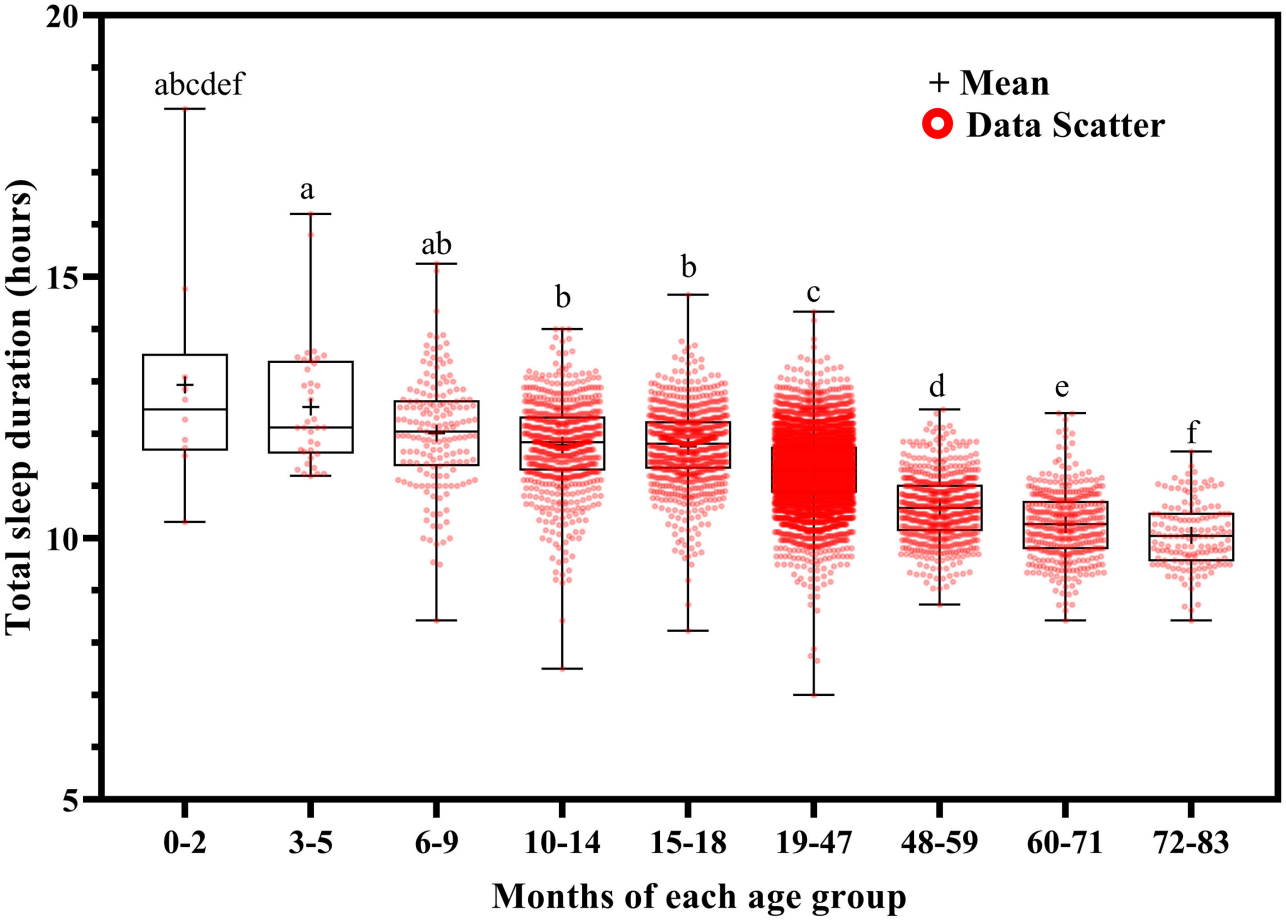
Results of the one-way ANOVA for total sleep duration stratified by age.

**Figure 7:**
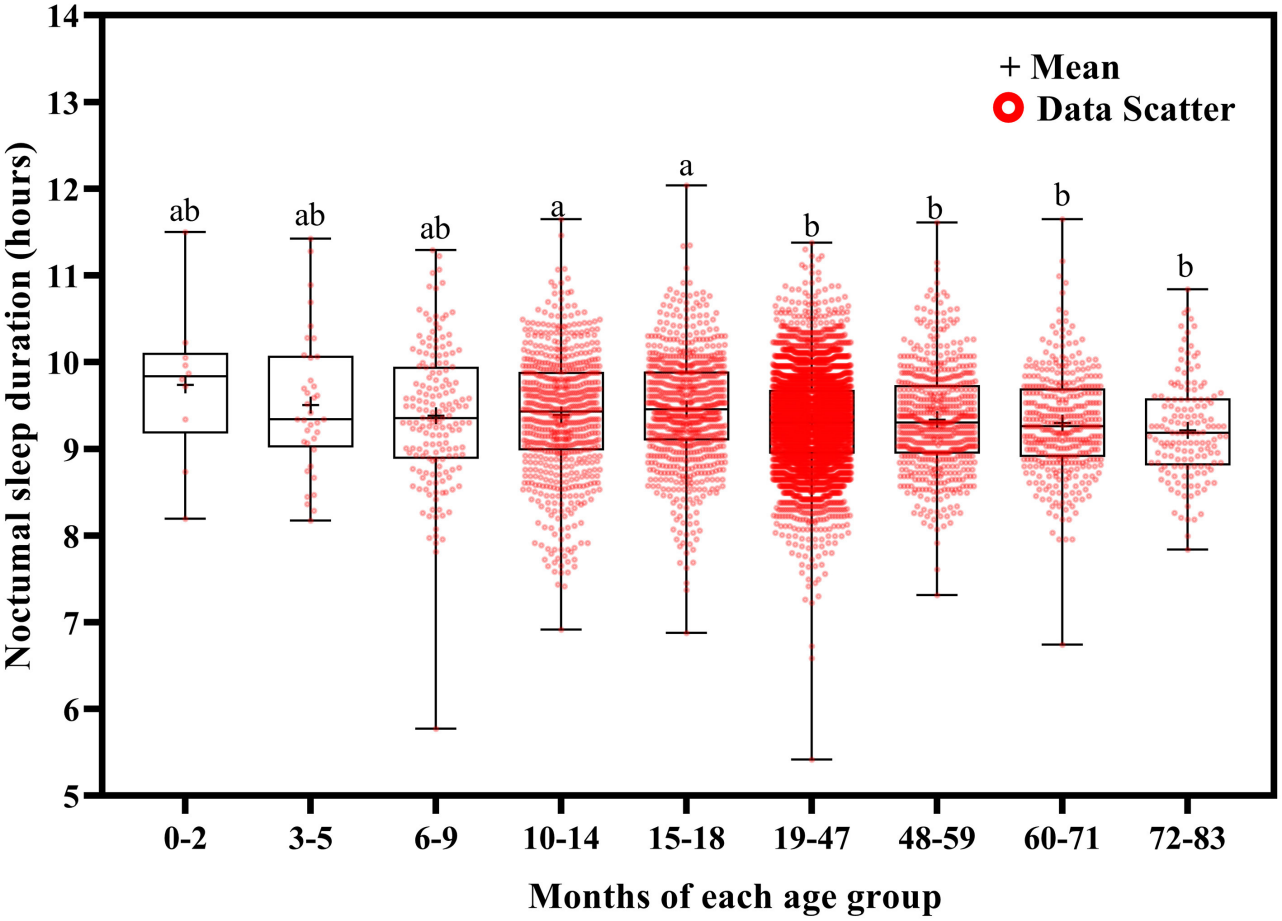
Results of the one-way ANOVA for noctumal sleep duration stratified by age.

**Figure 8:**
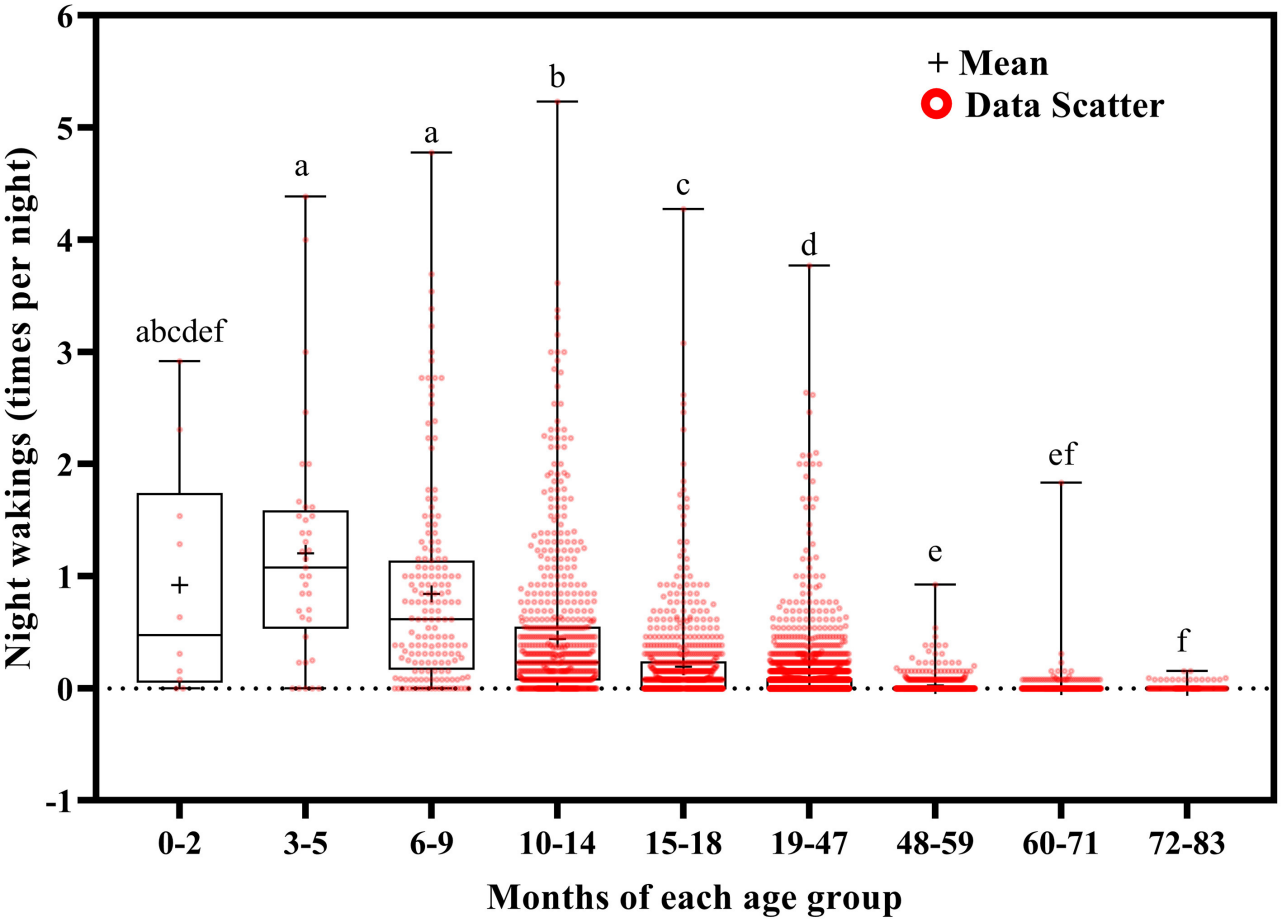
Results of the one-way ANOVA for night wakings stratified by age.

**Figure 9:**
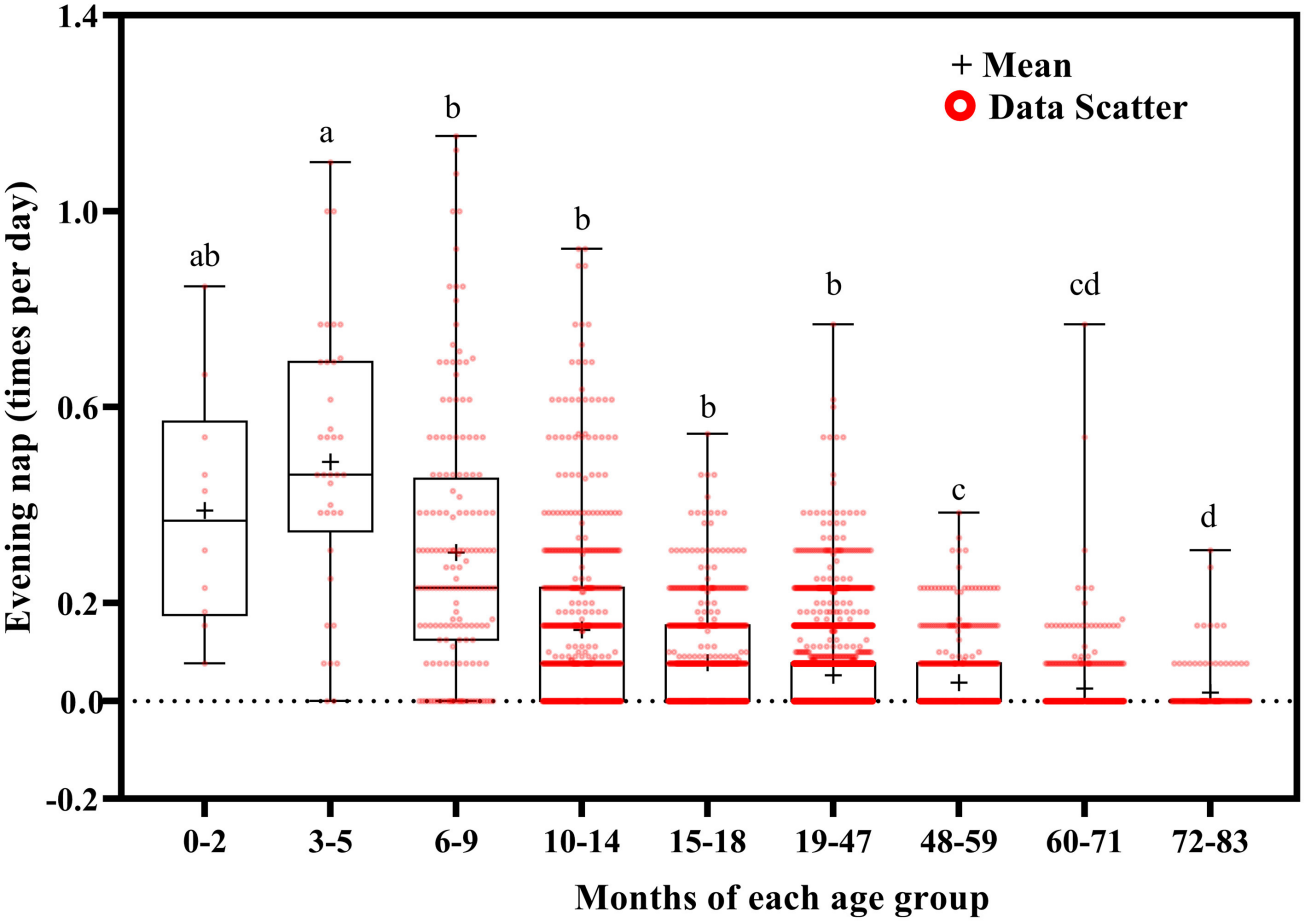
Results of the one-way ANOVA for evening nap stratified by age.

**Figure 10:**
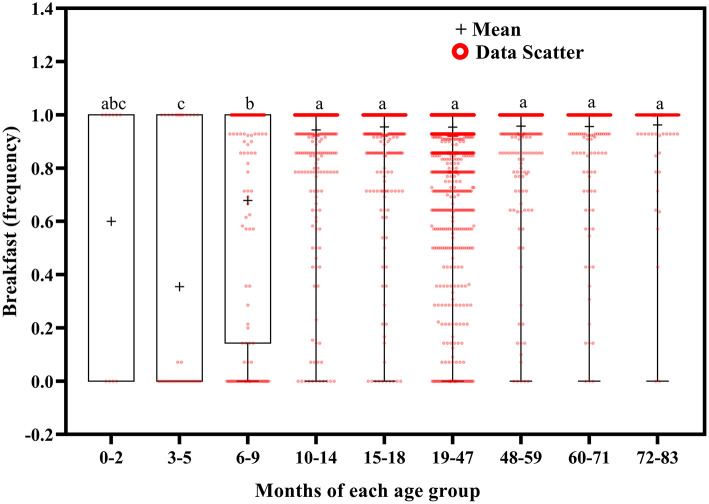
Results of the one-way ANOVA for breakfast stratified by age.

**Figure 11:**
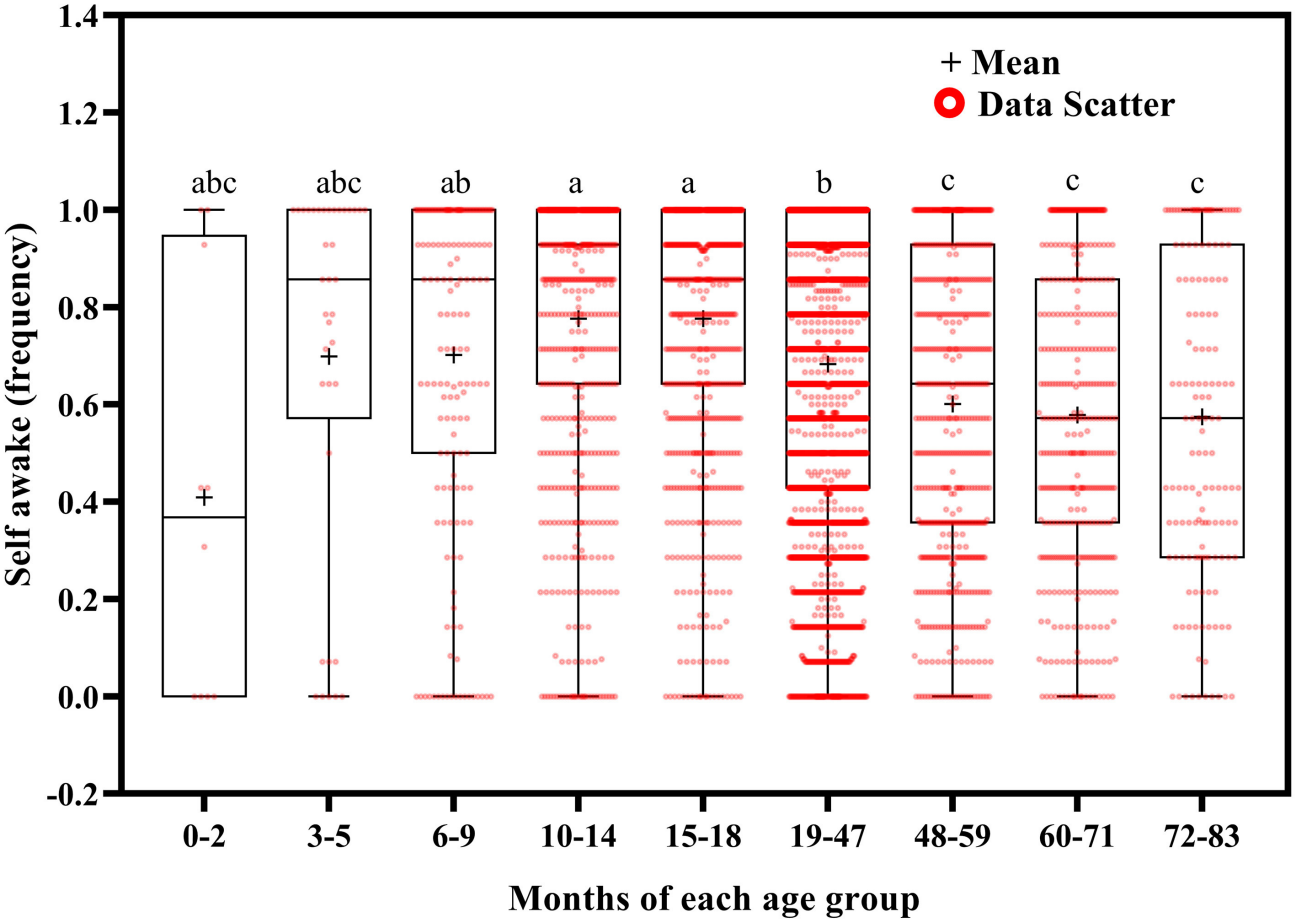
Results of the one-way ANOVA for self-awake stratified by age.

Using Welch's ANOVA with the Tamhene *post-hoc* test, we identified significant age-related differences in all of the variables in the 14-day sleep log (Table 2). Figures 2–11 show the box plots for each variable according to age using the alphabetical labeling method. If one or more of the alphabets in each group is the same, it means that there is no difference between the two groups. And if the alphabets are completely different, it means that there is a difference between the two groups.

**Table 2:** Results of Welch's ANOVA with Tamhene *post-hoc* testing for each variable.

**Groups**	**Months**	***N***	**Wake up time**	**Wake up time variation**	**Bed time**	**Bed time variation**	**Nocturnal sleep duration**	**Total sleep duration**	**Night wakings**	**Evening nap**	**Breakfast**	**Self-awake**
0	0–2 m	10	*F*_(8, 170.28)_ = 43.28 *p* <0.001 2, 3, 4 <5, 6, 7, 8; 3 <4	*F*_(8, 170.01)_ = 11.82 *p* <0.001 1 > 4, 5, 6, 7, 8; 2 > 5, 6, 7, 8; 3,4 > 6, 7, 8; 5 > 6, 7	*F*_(8, 170.18)_ = 69.88 *p* <0.001 2, 3, 4 <5, 6, 7, 8	*F*_(8, 170.44)_ = 9.52 *p* <0.001 2, 3, 4 <5, 6, 7, 8	*F*_(8, 170.13)_ = 103.76 *p* <0.001 3 > 8; 4 > 5, 6, 7, 8	*F*_(8, 170.13)_ = 378.06 *p* <0.001 2, 3, 4 > 5 > 6 > 7 > 8; 1 > 3, 4, 5, 6, 7, 8	*F*_(8, 174.11)_ = 103.76 *p* <0.001 1, 2 > 3, 4, 5, 6, 7, 8; 6 > 8	*F*_(8, 170.30)_ = 88.60 *p* <0.001 0 > 6, 7, 8; 1 > 2 > 3 > 4 > 5 > 6, 7, 8; 6 > 8	*F*_(8, 169.87)_ = 16.42 *p* <0.001 1 <2 <3, 4,5,6,7,8	*F*_(8, 170.33)_ = 32.81 *p* <0.001 2, 5 > 6,7,8; 3,4 > 5,6,7,8
1	3–5 m	37										
2	6–9 m	168										
3	10–14 m	687										
4	15–18 m	786										
5	19–47 m	4138										
6	48–59 m	626										
7	60–71 m	407										
8	72–83 m	149										

Wake up time (Figure 2) and bed time (Figure 3) tended to increase with increasing age. Wake up times were earlier at ages 6–9 months than at ages 19–71 (*p* <0.001) and 72–83 (*p* = 0.016) months, at ages 10–14 months than at ages 15–18 (*p* = 0.003) and 19–83 (*p* <0.001) months, and at ages 15–18 months than at ages 19–71 (*p* < 0.001) and 72–83 (*p* = 0.007) months. Children aged 19–83 months had later bed times than those aged 6–9, 10–14, and 15–18 months (*p* < 0.001).

Wake up time variation tended to decrease with increasing age (Figure 4). It was greater at ages 3–5 months than at ages 15–18 (*p* = 0.048), 19–47 (*p* = 0.029), 48–59 (*p* = 0.005), and 60–83 (*p* = 0.004) months; at ages 6–9 months than at ages 19–47 (*p* = 0.011) and 48–83 (*p* < 0.001) months; at ages 10–14 months than at ages 48–83 months (*p* < 0.001); at ages 15–18 months than at ages 48–71 (*p* < 0.001) and 72–83 (*p* = 0.032) months; and at ages 19–47 months than at ages 48–71 months (*p* < 0.001).

Bed time variation tended to decrease with increasing age (Figure 5). It was greater at ages 3–5 months than at ages 10–14 (*p* = 0.035), 15–18 (*p* = 0.024), 19–47 (*p* = 0.003), 48–59 (*p* = 0.004), 60–71 (*p* = 0.002), and 72–83 (*p* = 0.001) months; at ages 6–9 months than at ages 19–47 (*p* = 0.013), 48–59 (*p* = 0.032), 60–71 (*p* = 0.008), and 72–83 (*p* = 0.003) months; at ages 10–14 months than at ages 19–47 (*p* < 0.001), 48–59 (*p* = 0.013), 60–71 (*p* = 0.002), and 72–83 (*p* = 0.002) months; and at ages 15–18 months than at ages 19–47 (*p* < 0.001), 48–59 (*p* = 0.026), 60–71 (*p* = 0.004), and 72–83 (*p* = 0.006) months.

Total sleep duration (Figure 6) and nocturnal sleep duration (Figure 7) tended to decrease with increasing age. Total sleep duration was longer at ages 3–5 months than at ages 10–14 (*p* = 0.021), 15–18 (*p* = 0.014), and 19–83 (*p* < 0.001) months; at ages 6–18 months than at ages 19–83 months (*p* < 0.001); at ages 19–47 months than at ages 48–83 months (*p* < 0.001); at ages 48–59 months than at ages 60–83 months (*p* < 0.001); and at ages 60–71 months than at ages 72–83 months (*p* < 0.001). Nocturnal sleep duration was longer in children aged 10–14 months than in those aged 72–83 months (*p* = 0.029) and in children aged 15–18 months than in those aged 19–47 (*p* < 0.001), 48–59 (*p* = 0.005), 60–71 (*p* < 0.001), and 72–83 (*p* < 0.001) months. The number of night wakings tended to decrease with increasing age (Figure 8). Night wakings were more frequent at ages 3–5 months than at ages 10–14 (*p* = 0.002) and 15–83 (*p* < 0.001) months; at ages 6–9 months than at ages 10–83 months (*p* < 0.001); at ages 10–14 months than at ages 15–83 months (*p* < 0.001); at ages 15–18 months than at ages 19–83 months (*p* < 0.001); at ages 19–47 months than at ages 48–83 months (*p* < 0.001); and at ages 48–59 months than at ages 72–83 months (*p* < 0.001).

Evening nap frequency tended to decrease with increasing age (Figure 9). Evening naps were more frequent at ages 0–2 months than at ages 48–59 (*p* = 0.050), 60–71 (*p* = 0.040), and 72–83 (*p* = 0.034) months; at ages 3–5 months than at ages 6–9 (*p* = 0.018) and 10–83 (*p* < 0.001) months; at ages 6–9 months than at ages 10–83 months (*p* < 0.001); at ages 10–14 months than at ages 15–83 months (*p* < 0.001); at ages 15–18 months than at ages 19–83 months (*p* < 0.001); at ages 19–47 months than at 48–83 months (*p* < 0.001); and at ages 48–59 months than at ages 72–83 months (*p* = 0.001).

Breakfast frequency tended to increase with increasing age (Figure 10). Children aged 3–5 months ate breakfast less often than those aged 6–9 (*p* = 0.015) and 10–83 (*p* < 0.001) months, and children aged 6–9 months ate breakfast less often than those aged 10–83 months (*p* < 0.001).

Self-awakening tended to decrease with increasing age (Figure 11). Children self-awoke more frequently at ages 10–18 months than at ages 19–83 months (*p* < 0.01); at ages 6–9 months than at ages 48–59 (*p* = 0.027), 60–71 (*p* = 0.003), and 72–83 (*p* = 0.033) months; and at ages 19–47 months than at ages 48–71 (*p* < 0.001) and 72–83 (*p* = 0.005) months.

### Generation of a classifier to screen for severe sleep disorders

Discriminant analysis was conducted for each age group except Group 0 (0–2 months), whose sample size (*n* = 10) was insufficient for analysis. Judgment of the children's sleep quality (1 and 0) served as the dependent variable. The significant influencing factors determined *via* the stepwise method served as the discriminant variables. In the stepwise method, a variable that minimizes Wilks' lambda for the entire dataset is submitted at each step, and the maximum number of steps is 20. The identity of the classifier that discriminates between the presence and absence of severe sleep disorders in each age group is shown in Table 3.

**Table 3:** Results of the discriminant analysis stratified by age.

**Groups**	**Months**	**Eigenvalue**	**Canonical correlation coefficient**	**wilks' Lambda**	***X** ^2^*	***df***	***p***	**Discriminant variable(s)**	**Accuracy**
1	3–5 m	0.628	0.621	0.614	16.804	1	0.000	Bed time	97.30%
2	6–9 m	0.502	0.578	0.666	67.111	2	0.000	Wake up time variation; Bed time	88.70%
3	10–14 m	0.299	0.480	0.770	178.292	6	0.000	Night wakings; Total sleep duration; Bed time; Bed time variation; Wake up time; Wake up time variation	86.80%
4	15–18 m	0.298	0.479	0.770	204.112	4	0.000	Wake up time; Wake up time variation; Bed time; Bed time variation	89.30%
5	19–47 m	0.352	0.510	0.740	1246.358	6	0.000	Breakfast; Night wakings; Wake up time; Wake up time variation; Bed time; Bed time variation	88.10%
6	48–59 m	0.299	0.480	0.770	162.944	3	0.000	Total sleep duration; Wake up time variation; Bed time	88%
7	60–71 m	0.164	0.376	0.859	61.390	3	0.000	Night wakings; Total sleep duration; Bed time	71.30%
8	72–83 m	0.963	0.700	0.509	98.128	3	0.000	Total sleep duration; Nocturnal sleep duration; Bed time	80.50%

Group 1 (3–5 months) had a high canonical correlation coefficient (0.621), which indicates that the discriminant function can discriminate the children's sleep quality well. In addition, Wilks' lambda was significant (*p* < 0.001), indicating that the distance between severe and non-severe sleep disorders was sufficiently large. The significant discriminant variable identified by the stepwise method for Group 1 was the bed time. In a cross-validation test, the accuracy was 97.3%. The Fisher function coefficients are shown in Table 4.

**Table 4:** Fisher function coefficients of 3–5 months age group.

**Discriminant variable**	**y**_0_	**y**_1_
Bed time	41.685	37.154
(constant)	−492.019	−391.015

The values of y_0_ and y_1_ are calculated by substituting each discriminant variable(s). If y_1_ > y_0_, the judgment is 1, which means that the child does not have a severe sleep disorder. If y_0_ > y_1_, the judgment is 0, which means that the child has a severe sleep disorder and needs immediate intervention. The advantage of using the Fisher function instead of the standardized canonical discriminant function is that it allows direct calculation of the values of the variables obtained from the 14-day sleep log.

Group 2 (6–9 months), Group 3 (10–14 months), Group 4 (15–18 months), Group 5 (19–47 months), Group 6 (48–59 months), Group 7 (60–71 months), and Group 8 (72-83 months) also had a high canonical correlation coefficient (0.578, 0.480, 0.479, 0.510, 0.480, 0.376, and 0.700, respectively) and a significant Wilkes' lambda (all *p* <0.001).

The significant discriminant variables for Group 2 were bed time and wake up time variation; the accuracy was 88.7%. The Fisher function coefficients are shown in Table 5.

**Table 5:** Fisher function coefficients of 6–9 months age group.

**Discriminant variables**	**y**_0_	**y**_1_
Wake up time variation	24.341	19.184
Bed time	34.666	31.853
(constant)	−400.922	−337.346

The significant discriminant variables for Group 3 were night wakings, total sleep duration, bed time, bed time variation, wake up time, and wake up time variation; the accuracy was 86.8%. The Fisher function coefficients are shown in Table 6.

**Table 6:** Fisher function coefficients of 10–14 months age group.

**Discriminant variables**	**y**_0_	**y**_1_
Night wakings	21.095	20.517
Total sleep duration	48.222	48.774
Bed time	−42.915	−41.966
Bed time variation	20.686	17.265
Wake up time	80.051	77.281
Wake up time variation	4.665	7.182
(constant)	−1016.801	−967.353

The significant discriminant variables for Group 4 were wake up time, wake up time variation, bed time, and bed time variation; the accuracy was 89.3%. The Fisher function coefficients are shown in Table 7.

**Table 7:** Fisher function coefficients of 15–18 months age group.

**Discriminant variables**	**y**_0_	**y**_1_
Wake up time	−14.981	−13.263
Wake up time variation	22.049	14.206
Bed time	63.949	60.297
Bed time variation	−12.001	−5.334
(constant)	−667.184	−594.396

The significant discriminant variables for Group 5 were breakfast, night wakings, bed time, bed time variation, wake up time, and wake up time variation; the accuracy was 88.1%. The Fisher function coefficients are shown in Table 8.

**Table 8:** Fisher function coefficients of 19–47 months age group.

**Discriminant variables**	**y**_0_	**y**_1_
Breakfast	56.681	58.185
Night wakings	20.155	17.231
Wake up time	−13.828	−14.662
Wake up time variation	5.050	1.415
Bed time	80.974	77.699
Bed time variation	−14.396	−12.494
(constant)	−892.961	−814.159

The significant discriminant variables for Group 6 were total sleep duration, bed time variation, and bed time; the accuracy was 88%. The Fisher function coefficients are shown in Table 9.

**Table 9:** Fisher function coefficients of 48–59 months age group.

**Discriminant variables**	**y**_0_	**y**_1_
Total sleep duration	54.291	52.887
Wake up time variation	9.948	6.118
Bed time	89.612	85.382
(constant)	−1308.482	−1199.060

The significant discriminant variables for Group 7 were night wakings, total sleep duration, and bed time; the accuracy was 71.3%. The Fisher function coefficients are shown in Table 10.

**Table 10:** Fisher function coefficients of 60–71 months age group.

**Discriminant variables**	**y**_0_	**y**_1_
Night wakings	39.119	35.671
Total sleep duration	48.610	47.691
Bed time	77.240	75.409
(constant)	−1106.977	−1057.470

The significant discriminant variables for Group 8 were total sleep duration, nocturnal sleep duration, and bed time; the accuracy was 80.50%. The Fisher function coefficients are shown in Table 11.

**Table 11:** Fisher function coefficients of 72–83 months age group.

**Discriminant variables**	**y**_0_	**y**_1_
Total sleep duration	54.552	49.927
Nocturnal sleep duration	30.080	33.847
Bed time	111.102	108.746
(constant)	−1633.058	−1569.110

## Discussion

### Sleep characteristics of different age groups

Using a large dataset of children's sleep status, we found that total sleep duration and nocturnal sleep duration decreased with increased age. In line with this finding, the American Academy of Sleep Medicine recommends the following sleep durations for children: 12–16 h at ages 4–11 months, 11–14 h at ages 1–2 years, 10–13 h at ages 3–5 years, and 9–12 h at ages 6–12 years (20).

In our study, increases in age were consistently associated with decreases in wake up time variation and bed time variation. By the age of 36 months, some children no longer need a nap. After the age of 48 months, children have almost no night wakings or evening naps; in fact, a nap is no longer necessary at this age if the child sleeps for 10–11 h at night. Pediatricians attribute this phenomenon to the gradual establishment of sleep patterns in children aged over 19 months (21).

We also found that as the children aged, they went to bed and woke up at later times and were less able to wake up by themselves. Possible causes include late bed time and poor sleep quality. A previous study demonstrated that children who engage in watching television and playing with smartphones or tablets go to bed at later times than those who do not (22). Stress and long work hours in a competitive society with a large amount of information may account in part for the deteriorating sleep quality (23), a phenomenon that seems to reflect the lifestyle of a modern society.

In this study, children older than 10 months consumed breakfast almost every day. This finding suggests that by 10 months of age, most children have developed a pattern of eating three times a day and that, despite the later wake up time, parents always serve their children breakfast before sending them to a nursery school.

The generally accepted developmental patterns are considered as follows.

In order to survive, the body has a mechanism that prioritizes “eating” over “sleeping.” Therefore, sleep time was reduced when child was hungry. According to the Guidelines for Breastfeeding and Weaning Support (Revised 2019) published on the website of the Japanese Ministry of Health, Labor and Welfare (24), milk consumption by infants during one session increases with growth, with the frequency being ~every 3 h around 1–2 months of age and every 4 h around 3–5 months of age. Around 3–4 months of age, the body's mechanisms gradually change to correspond to the repetitive light-dark rhythm of day and night, and the tendency of continuous sleep increases. By 5–6 months of age, the child's gastrointestinal tract has developed the ability to digest and absorb food, and weaning can begin while monitoring the child's condition. The amount of milk they drink at one time may increase, and by 6 months of age, they will feed less frequently at night and sleep for approximately 6 h at a time, without waking up in the middle of the night. Around 6–7 months of age, baby teeth begin to erupt and the child develops the ability to chew. Weaning is completed by 9–10 months of age, and the child begins to develop a rhythm of eating three times a day. Eating well provides energy for daytime activities and helps them sleep well at night.

Infants do not sleep throughout the night, so naps during daytime is important. However, napping in an uncontrolled manner can be detrimental. If the child sleeps well at night, the nap will be in the form of one morning and one afternoon nap at 1 year of age and one afternoon nap after 1 year 6 months of age. By the age of 4 years, the child no longer needs naps. However, for children without a good rhythm, they may nap in small increments, and although they may feel a little more energetic after sleeping, they may continue this repetitive state: waking up for a short while, playing, and then going back to sleep. In the absence of proper sleep, this unhealthy pattern is repeated. Therefore, it is important to establish a daily rhythm early.

Taken together, our findings, which were obtained by accessing a large dataset, are in line with the general characteristics of children's sleep.

Regarding the change in discriminant variables by age, bed time was found to be a discriminant variable for all age groups considering that earlier bed time ensures longer sleep. Next, wake up time variation was found to be a discriminant variable in all age groups except those aged 3–5 months and 60–83 months. Because a regular daily rhythm can foster good sleep behavior, wake up variation is an important indicator in determining sleep quality. In the 10–47 months age group, bed time, wake up time variation, bed time variation, and wake up time were found as discriminant variables. After 10 months of age, children have completed weaning and are adjusting their life and circadian rhythm. Therefore, daily bed time, bed time variation, wake up time, and wake up time variation can be used to determine if daily rhythm is proper. However, since children in the 10–14 months age group frequently wake up during the night, night wakings is an important indicator, and total sleep duration should also be included as an indicator since increased night wakings can reduce sleep time. Therefore, for the 10–14 months age group, sleep quality should be assessed using a total of six variables: bed time, bed time variation, wake up time, wake up time variation, night wakings, and total sleep duration. In addition, children aged 19–47 months are more active during the day as their physical growth and motor skills develop. Breakfast guarantees energy for daytime activities, which leads to good sleep at night. Therefore, it is reasonable to assess sleep quality in the 19–47 months age group using a total of six variables: bed time, bed time variation, wake up time, wake up time variation, breakfast, and night wakings.

Since children after the age of 4 years no longer need naps if they are sleeping well at night, naps are also an important indicator to assess sleep quality. In this study, evening nap (15:00–19:00) was extracted from the 14-day sleep logs and is considered to interfere with nighttime sleep based on clinical findings. Therefore, it is necessary to assess the presence and hours of naps; total sleep duration, bed time, and wake up time variation were accepted as discriminant variables in the 48–59 months age group and were considered valid. In the 60–71 months age group, total sleep duration, bed time, and night wakings were found to be discriminant variables, and since wake up time variation was excluded from the discriminant variables in the 60–83 months age group, it can be inferred that children in this age group wake up at the same time almost every day. Therefore, bed time, total sleep duration, and night wakings are important indicators to assess sleep quality. However, the accuracy of the discriminant analysis for the 60–71 months age group was somewhat low, suggesting that the validity of the discriminant variables should be re-examined in future studies. In the 72–83 months age group, total sleep duration, bed time, and nocturnal sleep duration were found to be discriminant variables for children after the age of 6 years, because their life, circadian, and dietary rhythms have already been established.

According to the Sleep Guidelines for Health Promotion 2014 published by the Japanese Ministry of Health, Labor and Welfare (25), lack of sleep at night can lead to poor brain function, impaired concentration, attention, memory, and judgment, accompanied by daytime sleepiness, fatigue, inactivity, irritability, and anger. Accidents, injuries, and failures increase and cause low self-esteem. Furthermore, chronic sleep deprivation has been shown to increase the risk of diabetes, obesity, hypertension, and dementia. Therefore, good quality sleep, especially at night, is of utmost importance and is a prerequisite for healthy life.

### Situations in which the classifier can be used to screen for severe sleep disorders

Using a statistical model generated *via* discriminant analysis, we herein developed a classifier for screening severe sleep disorders in children. Our classifier allows us to quickly screen for, and thus immediately treat, severe sleep disorders on the basis of data contained in a 14-day sleep log. Its accuracy was almost 80% or higher for all age groups; thus, it is a valid screening tool. As an advantage, it can be used to simultaneously screen many cases. We assume that our classifier can be used in the situations described below.

#### Use by local governments in regular health checkups conducted for children

Despite the high prevalence of sleep disorders in the general population, healthcare utilization among patients with sleep disorders is relatively low. This finding, which is based on information in the Cerner Health Facts database, suggests that awareness of sleep disorders and access to medical care for sleep disorders are poor (26). In addition, previous studies suggest that primary care providers and pediatricians may underdiagnose sleep disorders in children (27, 28). Therefore, it is necessary to educate parents about the importance of sleep for children during the health checkups mandated by local governments. In Japan, local governments conduct health checkups for children aged 3–4, 9–10, 18 months, and 3 years. At that time, parents could be asked to record their children's sleep patterns for the 14 days preceding the checkup and to submit the log at the checkup; the classifier would then be applied. Parents would then be informed of their children's sleep problems and given instructions on how to manage their children's sleep and sleep hygiene; they would also be advised to visit a pediatrician if necessary. This procedure would facilitate early detection and treatment of sleep disorders in children.

#### Use by parents via an application

Parents, especially first-time parents, sometimes feel anxious and lonely because they cannot talk to anyone about their child-raising concerns. To aid parents, we are developing an application that uses artificial intelligence to enable consultations between parents and pediatricians, senior mothers from NPOs, etc. on child-rearing issues. Parents will be able to monitor the sleep quality of their children anytime and anywhere by installing the application on their smartphones. The application, which will include the classifier, will alert parents to their children's sleep problems and will encourage those in need to consult a pediatrician.

As a ripple effect, alleviating sleep disorders would be expected to alleviate postpartum depression in mothers and prevent child abuse. Studies showed that the quality of children's sleep significantly predicted the quality of maternal sleep, and they highlight the importance of screening for and treating pediatric sleep disruptions (29). Another study showed that significant differences in postpartum depression were found between mothers of children with and without significant sleep disorders (30). Mothers of children with sleep disorders have poorer sleep quality and are easily fatigued during the day and night, affecting their care of the child, feeding, and response to the child's sleep behavior. Persistent sleep disorders can lead to a series of long-term effects such as growth retardation and behavioral problems, which increase parenting stress and lead to emotional distress, creating a vicious cycle. Therefore, screening for severe sleep disorder in children and promptly providing intervention treatment and sleep hygiene guidance can help alleviate postpartum depression in mothers (31). In addition, parental depression is one of the risk factors for child abuse (32, 33). Therefore, alleviating maternal postpartum depression can help prevent child abuse.

In conclusion, we expect that the classifier developed in this study will facilitate the screening of children's sleep disorders in multiple situations. A previous study showed that early detection and treatment of sleep disorders aided the treatment of mental disorders (34); therefore, use of the classifier may help improve mental status. In the future, we will further improve the accuracy of the automatic classifier by incorporating machine-learning techniques.

## Data availability statement

The datasets analyzed in this study can be found at https://akachan.doshisha.ac.jp/forresearcher/bu/bu-about (Doshisha University Center for Baby Science Joint Usage/Research Center).

## Ethics statement

The studies involving human participants were reviewed and approved by Doshisha University. Written informed consent from the participants' legal guardian/next of kin was not required to participate in this study in accordance with the national legislation and the institutional requirements.

## Author contributions

Conceptualization, supervision, project administration, and funding acquisition: MK and SI. Methodology, software, data curation, visualization, and writing-review and editing: MK and MJ. Validation: MJ, MK, and SI. Formal analysis and writing-orginal draft preparation: MJ. All authors contributed to the article and approved the submitted version.

## Funding

This work was supported by JST COI Grant Number JPMJCE1307, and JSPS KAKENHI Grant Number 17K01923.

## Conflict of interest

The authors declare that the research was conducted in the absence of any commercial or financial relationships that could be construed as a potential conflict of interest.

## Publisher's note

All claims expressed in this article are solely those of the authors and do not necessarily represent those of their affiliated organizations, or those of the publisher, the editors and the reviewers. Any product that may be evaluated in this article, or claim that may be made by its manufacturer, is not guaranteed or endorsed by the publisher.
